# QUality improvement in primary care to prevent hospitalisations and improve Effectiveness and efficiency of care for people Living with coronary heart disease (QUEL): protocol for a 24-month cluster randomised controlled trial in primary care

**DOI:** 10.1186/s12875-020-01105-0

**Published:** 2020-02-14

**Authors:** Julie Redfern, Nashid Hafiz, Karice Hyun, Andrew Knight, Charlotte Hespe, Clara K. Chow, Tom Briffa, Robyn Gallagher, Christopher Reid, David L. Hare, Nicholas Zwar, Mark Woodward, Stephen Jan, Emily R. Atkins, Tracey-Lea Laba, Elizabeth Halcomb, Laurent Billot, Tracey Johnson, Timothy Usherwood

**Affiliations:** 1 Westmead Applied Research Centre, Faculty of Medicine and Health, The University of Sydney, PO Box 154 Westmead, Sydney, NSW 2154 Australia; 2 Western Sydney Local Health District, Sydney, Australia; 3 The George Institute for Global Health, Sydney, Australia; 4 Primary ageind Integrated Care Unit, South Western Sydney Local Health District, Sydney, Australia; 5 University of New South Wales, Sydney, Australia; 6 School of Medicine Sydney, The University of Notre Dame Australia, Sydney, Australia; 7 School of Population and Global Health, Faculty of Health and Medical Sciences, The University of Western Australia, Sydney, Australia; 8 Sydney Nursing School, Faculty of Medcine and Health, University of Sydney, Sydney, Australia; 9 School of Public Health, Curtin University and School of Public Health and Preventive Medicine, Monash University, Melbourne, Australia; 10 Faculty of Medicine, Dentistry and Health Sciences, University of Melbourne, Austin Health, Heidelberg, Australia; 11 Faculty of Health Sciences & Medicine, Bond University, Gold Coast, Australia; 12 The George Institute for Global Health, University of Oxford, Oxford, UK; 13 Centre for Health Economics Research and Evaluation, University of Technology, Sydney, Australia; 14 School of Nursing, University of Wollongong, Wollongong, Australia; 15 Inala Primary Care, Brisbane, Queensland Australia; 16 Department of General Practice and Westmead Applied Research Centre, Westmead Clinical School, Faculty of Medicine and Health, University of Sydney, Sydney, Australia

**Keywords:** Quality improvement, Primary care, Secondary prevention, Coronary heart disease, Data, Cardiovascular disease, Health services, Data linkage

## Abstract

**Background:**

Cardiovascular disease (CVD), including coronary heart disease (CHD) and stroke, is the leading cause of death and disability globally. A large proportion of mortality occurs in people with prior CHD and effective and scalable strategies are needed to prevent associated deaths and hospitalisations. The aim of this study is to determine if a practice-level collaborative quality improvement program, focused on patients with CHD, reduces the rate of unplanned CVD hospitalisations and major adverse cardiovascular events, and increases the proportion of patients achieving risk factor targets at 24 months.

**Methods:**

Cluster randomised controlled trial (cRCT) to evaluate the effectiveness of a primary care quality improvement program in 50 primary care practices (n~ 10,000 patients) with 24-month follow-up. Eligible practices will be randomised (1:1) to participate in either the intervention (collaborative quality improvement program) or control (standard care) regimens. Outcomes will be assessed based on randomised allocation, according to intention-to-treat. The primary outcome is the proportion of patients with unplanned CVD hospitalisations at 2 years. Secondary outcomes are proportion of patients with major adverse cardiovascular events, proportion of patients who received prescriptions for guideline-recommended medicines, proportion of patients achieving national risk factor targets and proportion with a chronic disease management plan or review. Differences in the proportion of patients who are hospitalised (as well as binary secondary outcomes) will be analysed using log-binomial regression or robust Poisson regression, if necessary.

**Discussion:**

Despite extensive research with surrogate outcomes, to the authors’ knowledge, this is the first randomised controlled trial to evaluate the effectiveness of a data-driven collaborative quality improvement intervention on hospitalisations, CVD events and cardiovascular risk amongst patients with CHD in the primary care setting. The use of data linkage for collection of outcomes will enable evaluation of this potentially efficient strategy for improving management of risk and outcomes for people with heart disease.

**Trial registration:**

Australian New Zealand Clinical Trials Registry (ANZCTR) number ACTRN12619001790134 (dated 20th December 2019).

## Background

Cardiovascular disease (CVD), including coronary heart disease (CHD) and stroke, is the leading cause of death and disease burden globally [1]. CHD accounts for the greatest single disease morbidity and nearly one fifth of all deaths, with around a third of these occurring in people who have prior CHD [2, 3]. With an aging population, and more people surviving initial events, the burden of CHD is increasing and is projected to rise from around 47 million disability-adjusted life years (DALYs) globally in 1990 to 82 million DALYs by 2020 [3]. However, despite international guidelines recommending secondary prevention [4–6], adherence, access and sustainability of their implementation is suboptimal. Use of evidence-based secondary prevention medications and lifestyle change both decline in the initial 6 months after an event [7] and continue to decline thereafter [8]. Therefore, improving post-discharge care through secondary prevention strategies (healthy living, adherence to medicines) is an international priority requiring innovative and efficient strategies that support better patient care [9, 10].

Collaborative quality improvement initiatives offer an efficient way to support and improve health service delivery on a large scale. These ‘purposeful efforts to secure positive change’ have become a focus of activity within the international healthcare environment [11]. In response to increasing health demand, the Institute for Healthcare Improvement (US) developed the Breakthrough Collaborative Quality Improvement Methodology to make rapid improvements in quality while reducing costs [12]. This approach offers a scalable model by targeting stakeholders to drive improvement, leveraging the collective power of sites working simultaneously on the same problem and using data to drive performance [12]. Collaborative methodology has been applied to a range of healthcare systems with demonstrated success in areas such as asthma [13], chronic heart failure [14] and compliance with healthcare standards [15, 16]. While such programs have been evaluated, evidence for their impact and effectiveness has only focussed on surrogate endpoints and more robust evidence is needed [17, 18].

In recent years, the expansion of technology has enabled the integration of automated data extraction, which has expanded opportunity for data-driven quality improvement [19]. For example, in primary care, practices can utilise their routinely collected data to inform practice-level decision-making and implementation of associated quality improvement [20]. As such, a focus on data-driven quality improvement has become a key element within contemporary literature outlining the building blocks of high performing primary care practices [21]. Current data shows that only 38% of Australian patients with CVD receive a government-funded chronic disease management plan from their primary care provider [22]. The aim of the QUEL (QUality improvement in primary care to prevent hospitalisations and improve Effectiveness and efficiency of care for people Living with CHD) study is to evaluate whether a data-driven quality improvement program implemented in primary care reduces CVD hospitalisations and improves CVD risk factors and medication adherence in patients with CHD over 24 months.

Specific objectives are to determine if a primary care practice-level, collaborative quality improvement program:

i. reduces the rate of unplanned CVD hospitalisations and major adverse cardiovascular events (MACE) at 24 months in patients with CHD;
ii. increases the proportion of patients who are prescribed evidence-based medications for CVD, are achieving national targets for risk factors (cholesterol, blood pressure (BP), smoking) and have an active Chronic Disease Management (CDM) or review plan in place and to;
iii. determine barriers and enablers associated with implementation of the quality improvement program.

## Methods/design

### Study design

**Fig. 1:**
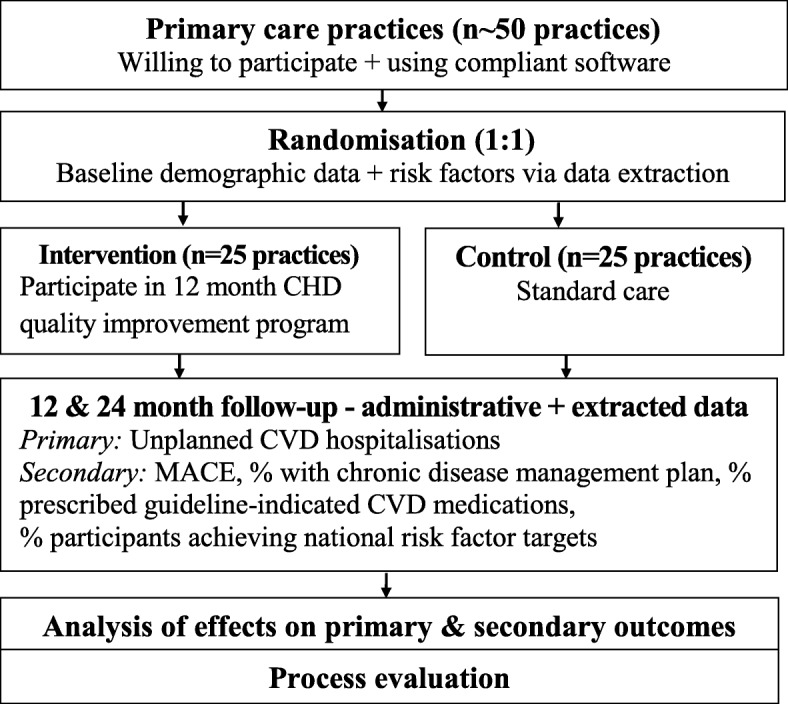
Flow diagram of QUEL cRCT

QUEL is a cluster randomised controlled trial (cRCT) with 1:1 randomisation recruiting 50 Australian primary care practices (n~ 10,000 patients with CHD) with 24-month follow-up to compare outcomes amongst practices allocated to collaborative quality improvement program versus standard care (Fig. 1). Data will be collected via linkage of routinely collected primary care practice data with administrative data (hospitalisations, deaths and pharmaceutical prescriptions).

### Practices and patients

Primary care practices will be identified through Australian Primary Health Networks (PHNs) across two states of Australia (New South Wales, Queensland). Research team members and representatives from the Improvement Foundation (an Australian not-for-profit organisation providing consultancy and training services in quality improvement) will approach all PHNs in participating states and provide information about the study. Supportive PHNs will then communicate with primary care practices in their jurisdictions to seek expressions of interest (EOI) for participating. Research team members will coordinate receipt of EOIs and contact practice staff to confirm eligibility and commence formal recruitment processes, signing of agreements and enabling automated data extraction from practice software. Inclusion and exclusion criteria for practice are detailed below.

Practices will be eligible to participate if they;

i. manage ≥100 patients year with prevalent CHD and
ii. use practice software that is compliant with Improvement Foundation systems (such as Medical Director, Best Practice, Communicare, Monet and Clarity which account for the majority of practice management software market-share in Australia).

Practices will be excluded if:

i. they are unwilling to provide written agreement to participate in the quality improvement program or
ii. the primary care practice is already participating in a formal quality improvement project targeting CVD.

The patient cohort for the QUEL will comprise a dataset of all eligible patients presenting to participating practices who meet the following inclusion criteria.:

i. are ≥18 years with a documented diagnosis of CHD in the primary care record of a participating practice, and
ii. have visited the participating practice at least once in the previous 12 months.

### Ethics

This study will adhere to the National Health and Medical Research Council (NHMRC) ethical guidelines for human research [23] and processes are detailed elsewhere [24]. Given this is a cRCT with a practice-level intervention, patient data will be extracted with a waiver of consent. Ethics approval has been obtained from the New South Wales Population & Health Services Research Ethics Committee (HREC, HREC/18/CIPHS/44), which also meets the formal approval requirements for Queensland. A waiver of consent is required because it will be impractical to collect individual consent from each patient and data linkage will be via data extraction from clinical software. The intervention will occur at the practice-level and it is not anticipated there will be any harm to individual patients. However, if a practice wishes to withdraw from the study they will be free to do so at any time. Any necessary protocol amendments will be approved by the ethics committee, investigators, trial registries and if required the journal should the need arise.

### Intervention and control groups

Practices allocated to the intervention will participate in a CHD secondary prevention collaborative quality improvement program. The program is designed to use data and practice level engagement to implement rapid and progressive changes based on Plan, Do, Study, Act (PDSA) cycles for pre-determined performance targets [21]. The program is underpinned by the psychology of behaviour change, active practice-level engagement combined with extraction and utilisation of electronically extracted data from practice software [12]. Practices will collaborate and support each other between and within practices in an ongoing way over 12 months to achieve a set of key performance measures (Table 1). These measures have been pre-determined based on guidelines [6] and availability of data for extraction and were developed and approved through an iterative process involving study investigators, clinicians and quality improvement experts. Intervention practices will receive their summarised performance data monthly.

**Table 1:** QUEL intervention key performance measures

• Proportion of patients with CHD where LDL has been recorded within the previous 12 months	
• Proportion of patients with CHD whose most recent LDL result was less than 2.0 mmol/L	
• Proportion of patients with CHD with a recorded BP reading taken within the previous 12 months	
• Proportion of patients with CHD whose most recent BP reading, taken within the previous 12 months, was less than or equal to 130/80 mmHg	
• Proportion of patients with CHD whose smoking status has been recorded	
• Proportion of patients with CHD recorded as a current smoker	
• Proportion of patients with CHD who are currently prescribed an anti-platelet agent	
• Proportion of patients with CHD who are currently prescribed a statin	
• Proportion of patients with CHD who are currently prescribed an ACE inhibitor or ARB	
• The proportion of patients with CHD with MBS Items 721 or 732 claimed (GP Management Plans or review)	
• Proportion of patients with CHD who have an influenza vaccination recorded within the previous 12 months	

*CHD* Coronary heart disease, *LDL* Low density lipoprotein, *BP* Blood pressure, Angiotensin-converting-enzyme (ACE) inhibitor, *ARB* Angiotensin II receptor blockers, *MBS* Medicare Benefits Scheme, *GP* General practice

The 12 month intervention will deliver the collaborative quality improvement program via (i) *Learning workshops* where a minimum of two practice staff (ideally one GP and one practice staff) participate in two online and two face-to-face learning workshops (based on Langley and Nolan model for improvement) [21]; (ii) *Activity periods* where practices use their own electronically extracted data to test and implement improvements through cycles of small step-wise changes; (iii) *Data reporting and feedback* where practices submit monthly data and PDSA cycles on which they are provided objective feedback (telephone and in-person visits) on their outcomes and progress and; (iv) *Transfer* where PHNs share learnings from practices within their jurisdictions [18]. Only personnel trained in delivery of collaborative quality improvement will deliver the practice support and all practices will have access to an online Sharepoint website for regular communication and support.

Practices allocated to the control group will participate in usual care without access to the quality improvement intervention for CHD during the study period. Control practices will be offered an opportunity to participate in a series of virtual workshops after data collection has closed. No individuals presenting to a participating practice (intervention or control) during the study period will be restricted in any way in terms of the care and treatment they receive from their healthcare providers. As this is a cRCT, it is not anticipated any post-trial care will be required for individual patients. However, after completion of follow-up, practices allocated to the control group will be offered the opportunity to receive support for quality improvement activities via a virtual program delivered by the Improvement Foundation. Their participation will be voluntary and will not impact on data collection.

### Outcomes

The primary outcome is the proportion of patients with unplanned CVD hospitalisations within 2 years of baseline data extraction (and commencement of intervention for those allocated to intervention group). For this study, CVD is defined as any condition involving the heart, brain or peripheral blood vessels and includes CHD (such as angina and myocardial infarction, MI), cerebrovascular disease (such as stroke), peripheral arterial disease and other conditions including heart failure and atrial fibrillation [25].

Secondary outcomes, also at 2 years) are;

i. Proportion of patients with major adverse cardiac and cerebrovascular events (fatal and non-fatal) that includes CHD (angina or MI), stroke or CVD death,
ii. proportion of patients who received guideline-recommended medicines,
iii. proportion of patients with a chronic disease management plan or review (Australian Medicare Item numbers 721 or 732 respectively) and;
iv. proportion of patients achieving national targets for CVD risk factors (total cholesterol, systolic blood pressure, smoking).

### Data collection and management

All data will be collected at baseline, 12 and 24 months. CVD hospitalisations and cardiovascular events will be collected via state-based administrative admissions data (depending on location of recruited practices). Individual patient deaths will be collected via linkage with the National Death Index and medication prescriptions and health service utilisation via linkage with the Australian Government Department of Health Pharmaceutical Benefits Scheme (PBS) and Medical Benefits Scheme (MBS) schemes respectively. Probabilistic matching will be used to link records and the estimated proportion of invalid and missed links using data linkage will be very low [26]. Data collection pertaining to the proportion of patients with chronic disease management plans and achieving national risk factor targets (cholesterol, smoking, and BP) will be electronically extracted from participating practice software systems using an automated data extraction tool with encrypted identifiers attached to patient data.

All data will be stored on a password-protected Secure Unified Research Environment (SURE), which is a purpose-built, remote-access data storage facility. This environment allows researchers to safely access, store and analyse study data [27]. Only aggregated and analysed data can be exported and data in SURE cannot be copied, downloaded or transmitted by email or other means. Only trained study research staff will have access to the SURE facility through a staff-specific username and password. The linked dataset will therefore be anonymised and the research team who will be analysing the data and delivering the intervention will not have access to individual-level data. This maximises privacy and forms an important aspect of the requirements for waiver of consent approval. Electronic files containing linked data for analysis will be stored on a Virtual Project Workspace within SURE and the access period shall be from study commencement for 7 years to enable all analyses to be completed. At the end of this data retention period, the tapes holding the archived data files will be physically destroyed.

### Randomisation

Practices will be randomised 1:1 to intervention (collaborative quality improvement program) or control groups using a computer-generated sequence generated with SAS 9.4 (Proc Surveyselect). Randomisation will be stratified according to two subgroups - rural versus urban location and size of the practice (≤2 versus > 2 GPs in a practice). The statistician performing randomisation will be blinded to practice names and details and only exposed to the practice characteristics that enable stratification. Once allocation is completed, a research team member will be provided the allocation list to enable communication with practices and commence arrangements for their respective requirements. It is not be possible to conceal the group allocation from the practices themselves or the research team delivering the intervention. However, given data is collected via linkage and not performed by research staff, it is essentially concealed. The statistician conducting analysis will be blinded to practice allocation.

### Sample size

The target sample size is 6050 (3025 per group), obtained from 50 practices (25 per randomised group) with an average cluster size of 121 patients per practice. This is estimated to provide 80% power to detect a ratio of the group proportions (or relative risk) of 0.75. This sample size assumes a control group readmission rate of 35% based on a recent Australian cohort study (*n* = 6172) reporting an atherothrombotic disease readmission rate of 35% at 2 years for patients with CHD [28]. The estimation assumes a significance level of 0.05 and an intra-class correlation coefficient (ICC) of 0.05. The ICC is based on data from two cross-sectional studies in Australian primary care [29, 30]. Loss to follow-up is anticipated to be very minimal given the primary outcome will be obtained via data linkage [26].

### Statistical analyses

Analyses will be conducted at the individual level while accounting for clustering of patients within practices. Intention-to-treat principle will be followed with patients analysed according to their randomisation group. Differences in the proportion of patients who are hospitalised (as well as binary secondary outcomes) will be analysed using log-binomial regression or robust Poisson regression in case of convergence issues. Clustering will be accounted for by modelling the correlation among patients from the same cluster using generalised estimating equations with an exchangeable correlation structure. Sensitivity analyses will include analyses of yearly rates using Poisson regression and/or time-to-event analyses via Cox models. Adjusted analyses will also be performed to account for baseline imbalances in cluster and patient characteristics. Pre-specified sub-group analyses will be used to determine the impact of the intervention on different patient groups (male v female; low SES v high SES, different CVD subgroups, influenza vaccination or not) and practice types (e.g., large v small and urban v rural). This will be done by adding the subgroup variable as well as its interaction with the intervention to the main analysis model. As data will be extracted from primary care practice clinical records and administrative data, if not recorded, it will be assumed that the task has not been done. A detailed analysis plan including mock tables will be developed and signed-off prior to unblinding.

### Barriers and enablers to implementation

This evaluation will enable examination of barriers and enablers to implementation of the quality improvement program. For example, level of support and expertise needed for practices to engage with the intervention as well as time spent preparing PDSA cycles and their delivery. Analyses will be informed by the Pawson and Tilley realistic evaluation model, which seeks to understand human choices and actions, within a systems context [31]. We will use a mixed methods approach with 3 data sources: (i) quantitative data related to practice engagement, attendance, time commitment, software capability, staff skills and capacity; (ii) survey of intervention practices to examine satisfaction and utility and (iii) semi-structured interviews with practice staff who participated in workshops and PHN representatives to identify capability and barriers and enablers to implementation. To obtain a broad range of views we will use maximum variation purposive sampling based on patient and practice characteristics [32]. Sampling will continue until thematic saturation is reached. Analyses (NVivo 11) will be thematic with coding based on emergent themes.

## Discussion

To the authors’ knowledge this is the first randomised controlled trial to evaluate the effectiveness of a data-driven collaborative quality improvement intervention in primary care on hospitalisations and events amongst patients with CHD. The use of data linkage for collection of outcomes will enable evaluation of this potentially efficient strategy for improving management of risk and outcomes for people with CHD. Dissemination plans at the conclusion of the study include a written report to all investigators, PHNs and practices involved in the study. In addition, the results will be submitted to a peer reviewed journal and presented at scientific conferences.

Evidence from a recent systematic review (64 studies) found that collaborative quality improvement promotes shared learning and clinical processes [33]. However, the authors conclude by highlighting that although results are encouraging the studies lack scientific quality and robust methodology. The QUEL study will overcome these limitations and the design has enabled collection of data for hospitalisations for a large population.

At the conclusion of this trial we expect to have evidence for a scalable solution to the evidence-practice gaps in secondary prevention of CHD. We will have determined the impact of the intervention on health outcomes (hospitalisations, proportion of patients with management plans and risk factor levels). We will also have rigorous data about program implementation in terms of barriers and enablers and we will have the first high-quality evidence in the world on the effectiveness and of implementing a collaborative quality improvement strategy. We will therefore be in a strong position to inform policy and to create an implementation plan.

## Data Availability

Not applicable.

## Abbreviations

ACE: Angiotensin-converting-enzyme
ANZCTR: Australian New Zealand Clinical Trials Registry
ARB: Angiotensin II receptor blockers
BP: Blood pressure
CHD: Coronary heart disease
cRCT: Cluster randomised controlled trial
CVD: Cardiovascular disease
DALYs: Disability-adjusted life years
EOI: Expressions of interest
GP: General practitioner
ICC: Intraclass correlation coefficient
LDL: Low density lipoprotein cholesterol
MACE: Major adverse cardiovascular events
MBS: Medical Benefits Scheme
MI: Myocardial infarction
NSW: New South Wales
PBS: Pharmaceutical Benefits Scheme
PDSA: Plan, Do, Study, Act
PHN: Australian Primary Health Networks
QUEL: QUality improvement in primary care to prevent hospitalisations and improve Effectiveness and efficiency of care for people Living with coronary heart disease
SES: Socioeconomic status
SURE: Secure Unified Research Environment
